# Two dynamic modes to streamline challenging atomic force microscopy measurements

**DOI:** 10.3762/bjnano.12.90

**Published:** 2021-11-15

**Authors:** Alexei G Temiryazev, Andrey V Krayev, Marina P Temiryazeva

**Affiliations:** 1 Kotel’nikov Institute of Radioengineering and Electronics of RAS, Fryazino Branch, Vvedensky Square 1, Fryazino 141190, Russia; 2 Horiba Instruments Inc., 359 Bel Marin Keys Boulevard, Suite 18, Novato, California 94949, United States

**Keywords:** atomic force microscopy, dissipation mode, scanning probe microscopy, vertical mode

## Abstract

The quality of topographic images obtained using atomic force microscopy strongly depends on the accuracy of the choice of scanning parameters. When using the most common scanning method – semicontact amplitude modulation (tapping) mode, the choice of scanning parameters is quite complicated, since it requires taking into account many factors and finding the optimal balance between them. A researcher’s task can be significantly simplified by introducing new scanning techniques. Two such techniques are described: vertical and dissipation modes. Significantly simplified and formalized choice of the imaging parameters in these modes allows addressing a wide range of formerly challenging tasks – from scanning rough samples with high aspect ratio features to molecular resolution imaging.

## Introduction

More than 30 years have passed since the introduction of atomic force microscopy (AFM) [1]. This technique has established itself as an indispensable tool for characterization not only in physics and chemistry, but also in related fields of research including medicine, biology, and materials science The relative ease of use of AFM and a large number of operating modes allowed for the study of mechanical, magnetic, and electrical properties of various objects. At the same time, surface profile measurements remain both the main application of the method and the basis of two-pass technics of magnetic and electrical measurements. In some cases, obtaining a good topographic image without artifacts and distortion is not an easy task. The operator must take into account a large number of often conflicting requirements, which leads to the need for very fine tuning of the scanning parameters, which, in turn, limits the successful imaging of complex samples only to experienced researchers. In this article, we would like to draw the attention of AFM practitioners to two operating modes, the vertical mode [2] and the dissipation mode [3–5], which can greatly simplify and expand the applicability of the AFM technique. In order to better understand the areas of applicability and benefits of the proposed methods, we will start with a brief overview of the standard scanning modes and their problems.

## Results and Discussion

### Basic AFM modes

A key element of a standard AFM is a probe, that is, a flexible console with a sharp tip at the end. Two main classes of scanning methods can be distinguished, namely contact and dynamic scanning. During contact scanning, the tip is pressed against the surface and the pressing force is controlled by the deflection of the console. A similar way to control the interaction of the probe with the sample is used in off-resonance dynamic modes [6]. Although they have various names, depending on the specific manufacturer (PeakForce Tapping, Hybrid Mode, Digital Pulsed Force Mode), a common feature of these methods is that the transition to the contact is carried out periodically with a frequency of 1–2 kHz. In addition to the surface profile, contact methods allow one to obtain some information on the mechanical properties of the material, provided that the contact area between the tip and the sample can be modeled with a reasonable degree of accuracy. In the case of the classical contact mode, the friction force can be measured; when using off-resonance dynamic modes, stiffness and adhesion in the samples can be determined. Obviously, in determining the mechanical properties, the force of tip–surface interaction should be somewhat greater than that required if the task is strictly limited to the measurement of topography.

When scanning in air, the interaction of the tip with the sample can be reduced, and the measurements themselves are performed more delicately when using resonance modes. In this class of AFM techniques, the probe is forced to oscillate close to its resonant frequency (usually 40–400 kHz). As the distance between the probe and the sample decreases, the oscillation amplitude *A* also decreases. A certain amplitude value is selected as a set point *A*_sp_ (a reference level). A feedback loop compares the current amplitude value with *A*_sp_ and, moving the *Z*-scanner vertically, maintains *A* = *A*_sp_. During scanning each line, lateral movement (in the *X* or *Y* direction) is performed at a constant speed *V*. This is the most common dynamic mode, which is called amplitude modulation (AM-AFM) [7] and has many other names (e.g., tapping mode or semi-contact mode). The common feature is amplitude feedback. An alternative method uses the resonant frequency of the probe as a feedback parameter and is called frequency modulation AFM (FM-AFM).

### Selection of scan parameters in amplitude modulation AFM

Let us consider which parameters we need to adjust in AM-AFM and what pitfalls are along the way there. In the amplitude modulation mode, scanning is performed with a constant excitation frequency and a constant power level supplied to the probe oscillation actuator. In a number of publications [8–10], it is proposed to drive at a frequency somewhat offset relative to the resonant frequency of the probe. Consideration of this issue is beyond the scope of this work. We will assume that, as in many AFM control programs, this parameter does not need to be selected, and the driving frequency is set equal to the resonant frequency of the probe located sufficiently far enough from the surface so that the van der Waals forces are negligible. The driving power is selected such that it provides a certain initial amplitude *A*_0_ of the probe oscillations (also far from the surface). This value is often called the amplitude of free oscillations. Next, we need to select a set point (reference) level *A*_sp_, we define it as *A*_sp_ = *p* × *A*_0_/100, that is, the amplitude during scanning will be *p* percent of the initial amplitude. In addition, we must set the scanning speed *V*. The problem is that the optimal choice of the values of *A*_0_*, p*, and *V* depends on the roughness of the surface to be investigated, information which we often do not have beforehand.

In general, to scan objects with high vertical protrusions or deep trenches/holes, it is necessary to increase the amplitude *A*_0_ and decrease the speed *V* [11]. Qualitatively, this can be understood from the following considerations. If there is a vertical wall of height *h*, then, at the moment when the probe quickly hits the wall, the oscillation amplitude decreases by the amount of *h*. The error signal δ*A* = *A* − *A*_sp_ appears, defined by the difference of the current amplitude and *A*_sp_. The value of δ*A* = −*h* is fed to the feedback system that separates the sample and the probe. When descending from the step, the opposite situation arises, that is, the amplitude increases and the feedback system has to reduce the distance between sample and probe. However, if the step is high and the initial amplitude is small, then the error signal is saturated. If *p* = 50%, then the maximum value of δ*A* does not exceed *A*_0_/2 and can be significantly less than *h*. This leads to inaccurate profile measurements. When descending from a step edge, so-called parachuting is observed and the actual topography profile is smoothed out. The climb to a step edge is also accompanied by an error in measuring the profile, and it often occurs also at zero amplitude, which means a pretty hard contact of the probe with the surface. This can lead to damage and contamination of the tip, as well as to the deformation of soft or poorly fixed objects on the sample. Errors in the measurement of the profile can be minimized by reducing the scanning speed. However, since the speed is constant, movement in flat areas will be unreasonably slow, which leads to a significant increase in the time required to obtain an image.

**Figure 1:**
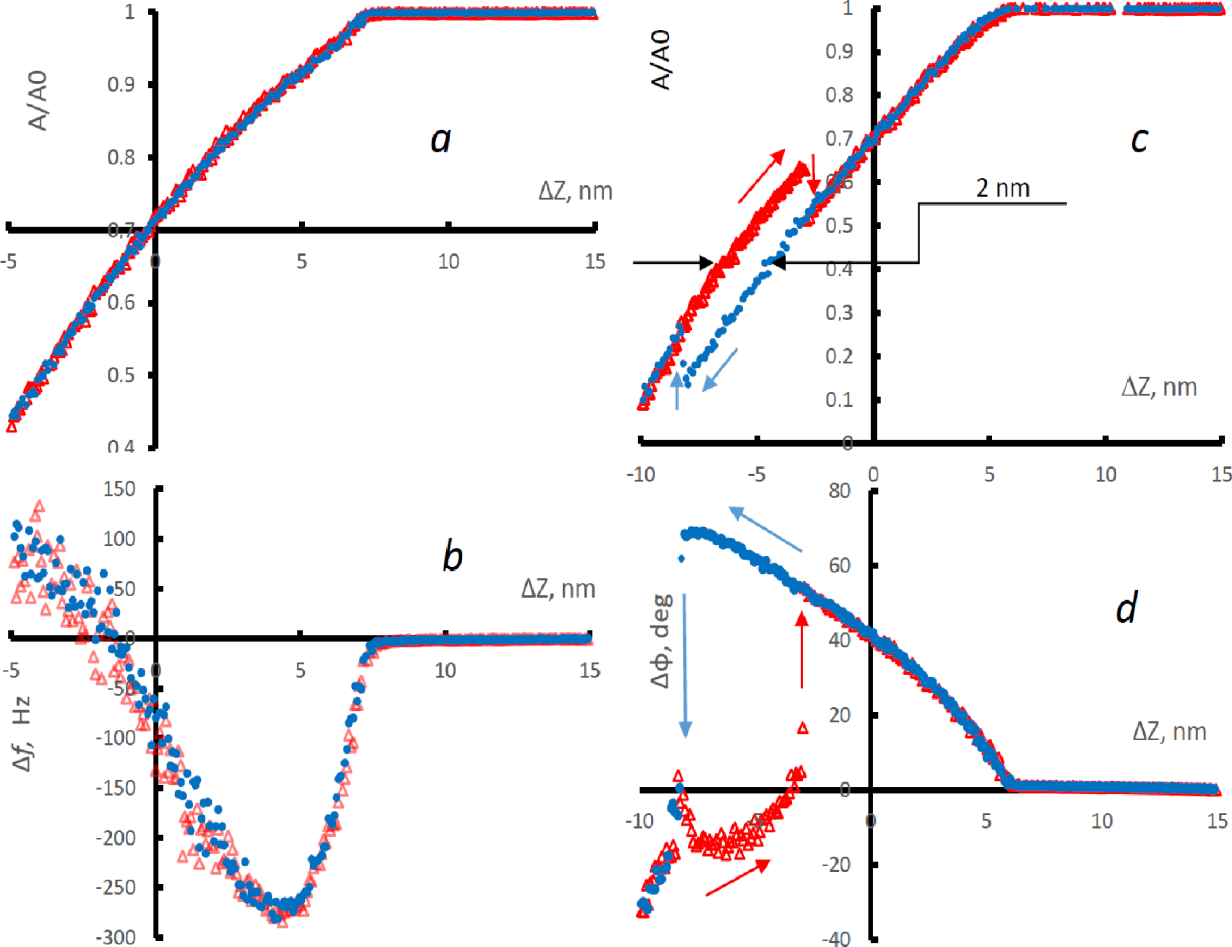
Approach (blue dots) and retraction (red triangles) curves. Change in amplitude (a) and frequency (b) during auto-tuning of the resonant frequency of the probe. Change in amplitude (c) and phase (d) at a fixed oscillation frequency of the probe.

One more factor affecting the quality of the resulting images should be noted. There are certain restrictions on the choice of parameters *A*_0_ and *A*_sp_. In the previous arguments, it was assumed that, through maintaining a constant amplitude of oscillations at the level *A* = *A*_sp_, we fix the distance *z* between the probe and the surface, that is, there is an unambiguous dependence of *A* on *z*. However, it is not always the case. As the probe approaches the surface, the decrease in amplitude occurs for two reasons. First, because of dissipation, and second, because the interaction of the probe with the surface leads to a change in its resonance frequency *f*_r_. This frequency shift is proportional to the force gradient and has a nonmonotonic dependence on *z* [4,12–14]*.* This dependence can be directly observed if resonance conditions are maintained, for example, if we use a phase-locked loop and constantly keep the driving frequency at resonance. As the probe approaches the surface, its resonant frequency first shifts downward under the action of attractive forces (e.g., van der Waals forces or capillary forces), and then increases when repulsive forces become dominant (Figure 1b). In AM-AFM, the driving frequency is fixed and is equal to the frequency of free oscillations of the probe. Any shift of the resonance will lead to a decrease in the amplitude of the forced oscillations even in the absence of dissipation. In this case, bistability is possible when both the negative and the positive shift of the resonant frequency correspond to the same amplitude [15]. The two possible resonant frequencies correspond to different distances between the probe and the sample. The condition under which the frequency shift is negative is called the net-attractive regime, and correspondingly, positive frequency shift is called net-repulsive regime. Switching from one regime to another is experimentally observed in the form of a sharp change in the phase of forced oscillations (Figure 1d). Hysteresis is often observed on the approach–retraction curve; switching of regimes occurs at different amplitudes depending on whether the probe approaches or retracts. There is a certain range of amplitudes within which the probe can be both in net-attraction and in net-repulsion (Figure 1c). Thus, under certain conditions, the unambiguous dependence between *A* and *z* is broken. If *A*_sp_ falls within this range, then due to the random switching of the interaction regime, pits (or protrusions) of about 1.5–2.5 nm height will appear in the obtained image of the surface. It is very important to select scanning parameters that guarantee the operation away from this region of bistability. The choice of a good value of *p* depends on the initial amplitude *A*_0_ and the tip sharpness. An increase in *A*_0_ and the use of the sharper probes leads to an increase in *p* at which the transition from attraction to repulsion occurs [16]. Qualitatively, this is easy to understand. The attractive forces for a blunt tip are greater because it has a larger effective area of interaction with the surface. In order for repulsion to become dominant against this background, more rigid contact with the surface is required, which is achieved with an increase in the initial amplitude *A*_0_ and/or a decrease in *A*_sp_.

We must decide in which regime (attraction or repulsion) the scan will be performed and accordingly select *A*_sp_. Factors to consider are the risk of tip wear, sample deformation, and tip contamination. The most important parameter here is the tip–sample force at the turn-around point of the oscillation cycle. Note that this force *F*_p_ can be repulsive even in net-attractive regime. In order to avoid tip wear and possible sample damage we need to keep the extreme force to a necessary minimum. There are different approaches for the choice of the best regime [17–21]. This selection is quite contradictory as the attraction regime provides the most delicate conditions that guarantee the preservation of the sharpness of the probe [22]. At the same time, if the probe is initially sharp, then this regime requires a very small initial amplitude (*A*_0_ is less than 10–20 nm), which, as noted above, can lead to damage to the probe if it bumps into a steep high wall.

Another important point. For high quality images, a correct feedback gain setting is required. We will not consider this issue in detail, since it is sufficiently general for all systems with negative feedback; the specificity associated with the operation of a scanning probe microscope can be found in [11]. We only note that errors in choosing this parameter can lead to image distortion and noise, as well as damage to the probe.

It can be seen from the foregoing that the choice of scanning parameters depends on both the characteristics of the sample and the sharpness of the probe. Optimization, as a rule, requires a test scanning and some experience with the device. To facilitate the work of the researcher it was proposed in [23] to use a special program for tuning, the algorithm of which involves the selection of parameters during the test scanning. In our paper, we would like to propose an alternative approach related to changing the scanning procedure itself.

### Vertical mode

The vertical mode (VM) is similar to amplitude modulation, that is, the probe oscillates near the resonant frequency, the driving frequency and power of the piezoelectric transducer are fixed. A key element of VM is a complete decoupling of lateral (*XY*) and vertical (*Z*) movement of the probe. We set two levels of the oscillation amplitude: a reference value *A*_1_ = *p*_1_ × *A*_0_/100 and the top value *A*_2_ = *p*_2_ × *A*_0_/100, where *A*_2_ > *A*_1_. The height is recorded at each point at the moment when the amplitude reaches the value of *A*_1_, as the probe moves vertically at a constant speed towards the surface. After the *A*_1_ level is reached and the value of corresponding height is stored, the probe moves away from the surface until the amplitude exceeds *A*_2_. Only upon reaching this condition, the probe starts lateral movement, that is, the transition to the next point. If during this movement the amplitude drops below the level *A*_2_, the lateral movement stops, the probe rises above the sample again until the amplitude exceeding *A_2_* is restored, after which the lateral transition resumes. Thus, at each measurement point, the amplitude is initially greater than *A*_2_, and in order to reach the *A*_1_ level, it is necessary to bring the probe closer to the surface.

Let us consider the main advantages of this method. One is the adaptive scanning speed. The levels of *A*_1_ and *A*_2_ may be very close. Their difference should only be much higher than the noise level. In our measurements, *p*_2_ − *p*_1_ = 4%, which for an initial amplitude *A*_0_ = 20 nm corresponds to a vertical displacement of less than 1 nm. This means that scanning a flat surface can be reasonably fast. However, if the probe encounters a tall (deep) feature, the imaging algorithm will provide additional time to trace steep topography. Likewise, when descending a steep edge, parachuting will not happen, as the probe will remain over each point as long as necessary to reach the reference level of the amplitude. Thus, the actual time taking to acquire a scan is determined both by the speed of movement between the adjacent points and the roughness of the sample. In many cases, the latter factor will predominantly determine the overall imaging time.

There is no need to adjust the feedback and it is possible to scan rough surfaces at small oscillation amplitude. Since feedback is not used in its regular sense, we have no restrictions associated with the need to increase the amplitude of oscillations when scanning objects with a large difference in height. One of the most important consequences of the imaging algorithm proposed above is the ability to keep the sharpness of ultrasharp probes when scanning rough surfaces and large areas. For any tip, the amplitude *A*_0_ can be chosen so small that the reference amplitude *A*_1_ at *p*_1_ = 70–80% corresponds to the net-attractive regime, which is the safest one for the probe. For a sharp tip, *A*_0_ is expected to be fairly small, less than 5–10 nm. Nevertheless, the VM allows for scanning a large rough area with a probe oscillating at a low amplitude. In contrast, the use of ultrasharp probes allows for performing extremely high-resolution imaging (down to molecular resolution) when scanning small and relatively flat areas. Thus, with the same probe, we can pre-scan a large area, find specific locations and perform high-resolution scans. The technique of high-resolution imaging will be discussed in the next section.

The VM avoids image artifacts associated with a sticking probe. In some cases, a sticking effect can be observed, when in the process of AFM imaging the amplitude of the probe oscillations sharply drops to zero [24]. This phenomenon is usually caused by a combination of small oscillation amplitude, inappropriately low spring constant of the probe (and thus too low energy stored in the vibration), strong attractive forces caused by some surface layers (water/hydrocarbons), and increased adhesion caused by the large contact area of a blunt tip. Regular AM-AFM feedback operation in the presence of sticking is very unstable and inhibits one from obtaining correct surface profile. Using the VM can help in this case. At each measurement point, we take a height measurement when the amplitude decreases to level *A*_1_. A further decrease in the amplitude is no longer relevant, since, according to the algorithm, the probe will be retracted from the surface at a distance that guarantees the restoration of oscillations.

The sticking effect can also be observed in the case of scanning samples with high aspect ratio features. In AM-AFM, when scanning such samples at low oscillation amplitude, the lateral displacement velocity *V* must be kept very low to minimize error. When approaching a steep (high or deep) object, the sensor begins to touch it with the side of the needle cone. The contact area increases sharply, sticking occurs and the amplitude drops to zero. Consequently, feedback retracts the probe, the amplitude is restored to a high level, the probe approaches the surface again, and the process is repeated. At a low scanning speed *V*, this leads to artifacts, that is, several periods of height fluctuations against the wall are recorded. In the VM, sticking can also occur, but it does not affect the measurement result.

Let us consider which parameters, in addition to *p*_1_ and *p*_2_, are important for the VM. In AM-AFM, it is sometimes suggested that one can use the return scan line to compensate for direct pass errors. When using the VM, the height measurement occurs during vertical movement, so it does not matter from which side the probe approaches the measurement point. It makes no sense to make measurements on the return pass. The scanning time can be reduced due to a faster return pass performed when the probe is retracted a certain distance from the surface. Thus, we introduce *T*_line_, the time of lateral moving at each line. It will consist of two parts: *T*_dir_, the time for lateral moving during the measurements (direct pass) and *T*_ret_, the time for the return pass. The time of vertical movements is not known in advance. It depends on the sample roughness and two parameters, namely the speed *V*_down_ of the probe approaching the surface at the point of measurement and the retraction speed *V*_up_. The time required to obtain a scan, *T*_scan_, will be the sum of the lateral and vertical movement times on all lines, as well as the ascent and descent times when switching the direct and return passes. Next, we enter two levels of height. A bottom level *H*_bot_ limits the depth at which the probe must reach the surface. If, during the measurement, at *H*_bot_ the oscillation amplitude still exceeds *A*_1_, we will write down all the necessary parameters and start moving to the next point. This allows us to continue scanning even if we cannot reach the bottom surface in the case of tall objects or samples with deep pits. Conversely, if at the top level *H*_top_ the oscillation amplitude is less than *A*_2_, we stop scanning. This prevents damage to the tip. Then, we need to mechanically move the entire *Z*-scanner to increase the distance to the sample.

**Figure 2:**
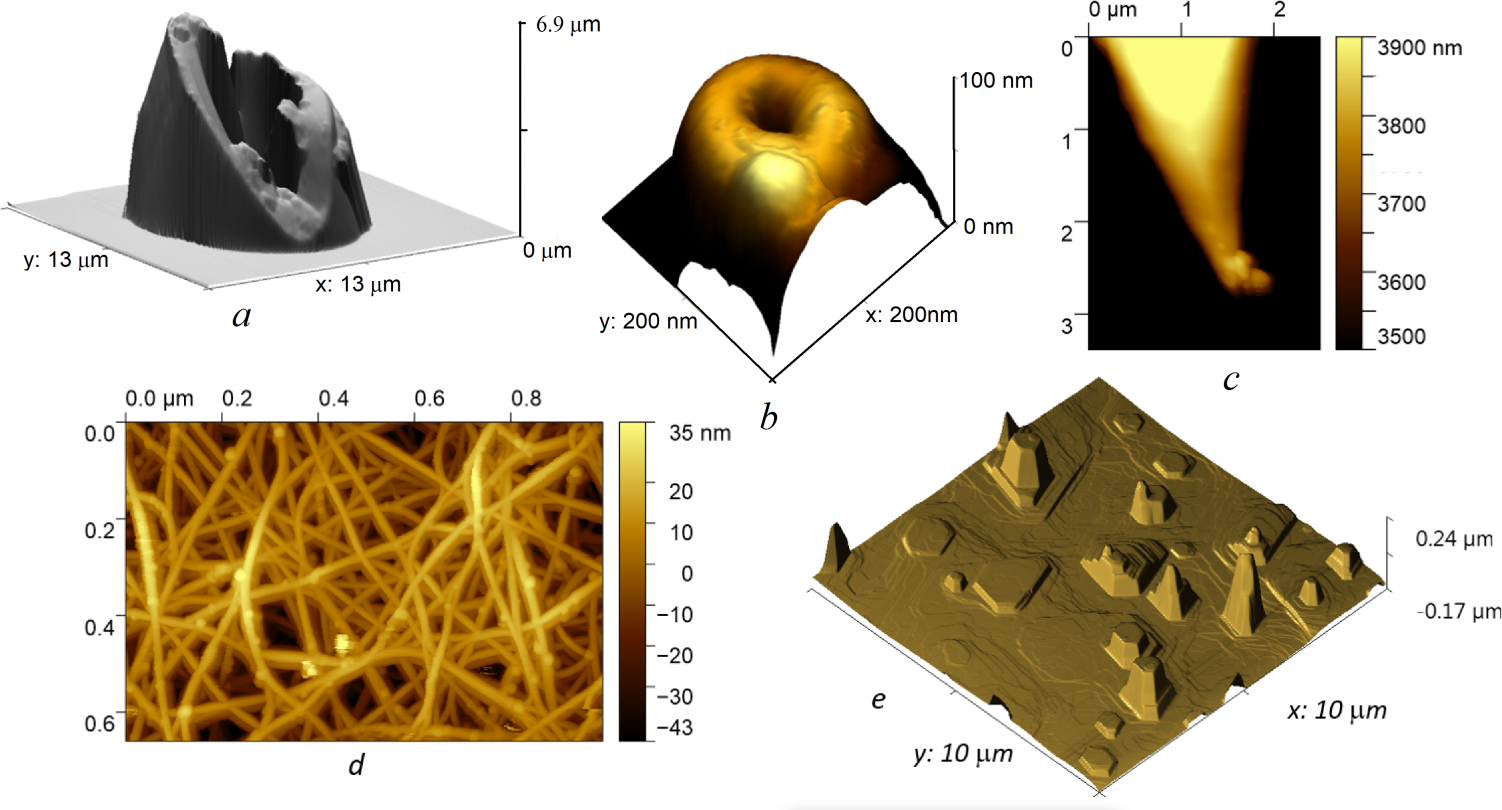
Vertical mode imaging. AFM images of the top of a broken micropipette (a) and an intact micropipette (b); AFM image of the tip of the probe (c), the surface of a SWCNT film (d); AFM image (without processing) of the surface of bismuth telluride (e). Scanning parameters for (a): 256 by 256 points, *A*_0_ = 12 nm, *T*_line_ = 2 s, *T*_dir_ =1.8 s, *V*_up_ = 20 µm/s, *V*_down_ =10 µm/s, *p*_1_ = 70%, *p*_2_ = *p*_1_ + 4%, *f*_r_ = 245 kHz, *T*_scan_ = 16 min. Scanning parameters for (d): 512 by 314 points, *A*_0_ = 12 nm, *T*_line_ = 1 s, *T*_dir_ = 0.8 s, *V*_up_ = 5 µm/s, *V*_down_ =2 µm/s, *p*_1_ = 70%, *p*_2_ = *p*_1_ + 8%, *f*_r_ = 233 kHz, *T*_scan_ = 21 min. Parameters for (e) are given in Supporting Information File 1.

Figure 2 presents some examples of scanning. The VM allows you to scan objects with high vertical walls without artifacts caused by parachuting and sticking (Figure 2a). Figure 2a and Figure 2b show the top of a micropipette, which is a probe in scanning ion conductance microscopy [25]. From Figure 2b, we can estimate that the diameter of the intact micropipette at its most protruding part is about 100 nm. To do this, we need to make a small scan about 200 nm in size. Even finding the right spot for this scan is a tricky task for standard AFM modes because micropipettes are much thinner than the AFM tip cone. To find it, you need to scan an area of several micrometers and be ready to encounter an object whose height exceeds the range of the *Z*-scanner. This task is easy to fulfil in the VM. If the probe hits something, it stops lateral movement and moves vertically to find the top of the object. If the object is too high and the *H*_top_ level is reached, scanning stops; this prevents damage to both the probe and the sample. In the case of a micropipette, a rough scan of a large area gives an image of the probe itself (see Supporting Information File 1). To see the end of the micropipette, we should select a small area near the highest point.

Figure 2c demonstrates the scan of the AFM tip. We chose contrast to show the end of the tip and to visualize the contamination there. Such a sample is available in any AFM laboratory and allows one to demonstrate some of the features that make it difficult to work in standard modes. On the one hand, next to the scan area, there is a high console of the cantilever; on the other hand, there is no bottom; the sample seems to be hanging in the air. When using VM, we limit the scan depth by setting *H*_bot_ (which is about 4 μm as seen on the height scale) and the scan will stop if the probe touches the console.

The VM was elaborated as a tool for imaging samples with high aspect ratio features (deep cavities or vertical walls). Our experience with VM has shown that this is a versatile mode, effective for scanning any objects. The point is that the VM allows using low amplitudes for scanning samples of any roughness. The ability to scan rough surfaces using a small oscillation amplitude and sharp probes in the attraction regime means that the contact area is small and the tip–sample interaction is negligible. On the one hand, this allows for high-quality imaging of loosely attached and soft objects, even if they have a high aspect ratio. For example, Figure 2d shows a film of single-walled carbon nanotubes (SWCNTs) [26]. This is very soft object that can be easily deformed by AFM probe. On the other hand, such a regime significantly reduces the likelihood of tip contamination. AFM image artifacts are often caused by a particle sticking to the tip (see Figure 2c). This not only degrades the image resolution. It also leads to the appearance of stripes in the image since the particle is poorly fixed and it may fall off or change its position during the scanning process. As a result, adjacent scan lines differ slightly in height. Such a defect is usually corrected by fitting the lines during subsequent image processing. In some cases, such as when high protrusions are adjacent to flat areas, this fitting will give poor results. Using VM yields high-quality raw images that do not require post-processing. Figure 2e and Figure S2 in Supporting Information File 1 illustrate this statement by the example of scanning the surface of bismuth telluride [27].

**Figure 3:**
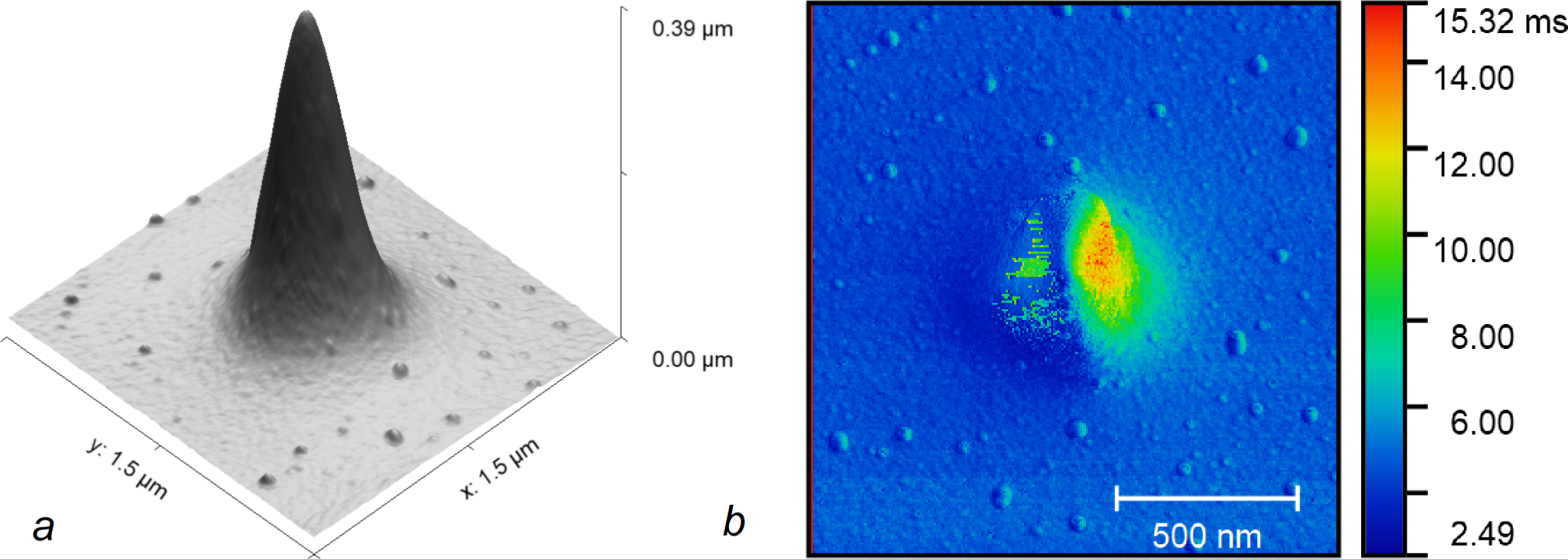
AFM image of the tip on a test grating TGT1 (a); time of the measurement at a point (b).

The main disadvantage of VM is the longer image acquisition time *T*_scan_ compared to standard scan modes. This is true if the surface is flat or if hard tapping at high initial amplitude is used for scanning a rough surface in AM-AFM. However, it might be discussed if we want to maintain the sharpness of the probe and are forced to scan with low *A*_0_. As an example, we use a sample that is easy to scan in any mode, namely a tip on a test grating TGT1 (NT-MDT). Figure 3a shows the image of the tip obtained using the VM with the following parameters: 256 by 256 points, *A*_0_ = 10 nm, *T*_line_ = 0.5 s, *T*_dir_ = 0.4 s, *V*_up_ = 5 µm/s, *V*_down_ = 2 µm/s, *p*_1_ = 70%, *p*_2_ = *p*_1_ + 4%. We used a Mikromasch NSC15 cantilever with a nominal force constant 46 N/m and resonance frequency of 328 kHz. This scan took *T*_scan_ = 6 min. Figure 3b shows the time spent on the measurement at each point. At flat areas, it is about 4 ms including 1.6 ms for the lateral movement from point to point. Thus, we spend an additional 2.4 ms for vertical movement. At the side of the tip, this time increases fivefold. This ensures an accurate measurement. Scanning in the standard AM-AFM mode (see Supporting Information File 1 for details) at the same value of *A*_0_ took 13 min at 0.33 Hz scan rate. The recorded tip shape was almost the same but there is a big difference in variation in the oscillation amplitude, which is a feedback error. The amplitude varied in the range of 15.4–16.7 relative units in VM and in a seven times wider range of 12.5–21.8 in the AM-AFM. This example shows that the VM can give better results even with shorter acquisition times.

We also note that in the VM, along with the measurement of height, the phase of probe oscillations can be recorded. The phase contrast obtained in this case, as in the case of AM-AFM, allows one to probe the heterogeneity of the mechanical properties of the surface [28], but it is free from artifacts associated with the influence of the surface profile on the feedback error (phase change at the step boundaries). Thus, in comparison with AM-AFM, the loss of the scanning speed in the VM is compensated by the gain in the ease of the AFM set up and the overall time required for collecting a high-quality image. There is no need for a test scan, it is easier to process collected images; due to the dramatic reduction of tip contamination, the probes do not have to be exchanged as frequently as in conventional AM-AFM imaging protocols. Easy and quick setup of the VM is of particular importance. Usually, it is enough to choose just one parameter, that is the initial amplitude of the oscillations. All other parameters, at this stage, can have fixed values. For example, we can use *p*_1_ = 70–80%, *p*_2_ = *p*_1_ + 4%, *T*_line_ = 1 s, *T*_dir_ = 0.8 s, *V*_up_ = 5 µm/s, *V*_down_ = 2 µm/s. Such a set will provide high-quality images of various samples. Further optimization of the parameters may be needed to address specific goals, for example, to reduce the scanning time. The selection of the value of initial amplitude cannot be avoided since it depends on the sharpness of the probe. If the measurements are to be conducted in the net-attractive regime (optimal from the point of view of the probe apex preservation), then for sharp probes the amplitude should be rather small, for example, 5–10 nm. If the probe is blunter, at low amplitudes the probe may stick, which will significantly increase the scanning time. In this case, we can increase initial amplitude, while remaining in the net-attractive regime. It is important that the choice of *A*_0_ is based on the approach curve; it is independent of surface topography and can be done prior to starting the actual scan. We used different types of cantilevers with a force constant in the range of 0.5–50 N/m and resonant frequencies of 40–400 kHz. Some of the probes had sharp custom-made spikes grown according to [29].

It should be noted that the VM is different from the jumping mode (JM) proposed in [30]. First, JM is a contact mode. Second, in jumping mode it is assumed that after measuring the height at a certain point, the probe is lifted by a certain distance, for the purpose of its safe transfer to the next measurement point. The VM does not imply separation with the surface, the probe is within the range of van der Waals forces almost all the time. Due to this, the scanning speed in flat areas increases significantly. We can consider the VM as a kind of feedback that during lateral movement does not track the height but only ensures that the probe does not get too close to the surface. Compared to the off-resonance dynamic modes, the main difference of the VM is that it is a resonance mode, which allows for a more gentle scanning in air than contact modes.

### Dissipation mode

It is commonly known that the lateral resolution of AFM images depends on the sharpness of the probe. A typical value of the radius of the tip curvature *r* is 10 nm. Specially made probes with carbon spikes have values of *r* about 2 nm [31–32]. Nevertheless, under certain conditions, for example, when measuring under high-vacuum conditions, the AFM allows for obtaining atomic resolution [33]. Such measurements are usually carried out in the frequency modulation mode, which means that the driving frequency is automatically kept equal to the current resonant frequency of the probe, and the shift of the resonant frequency is used as the set point. Historically, FM-AFM is commonly called non-contact atomic force microscopy [33]. However, repulsive forces are required to obtain high resolution, which implies some level of contact [34]. Qualitatively, this is easy to understand from the following reasoning. If a probe with a radius of curvature of the tip *r* touches a relatively smooth surface, then the contact area may have lateral dimensions substantially smaller than *r*. This will give a good resolution. With an increase in pressure, the contact area will increase, and the resolution will decline. Thus, in order to achieve high resolution, a precise adjustment of the scanning parameters is required that will allow for an operation within a narrow range of forces. The goal of optimizing the scan parameters is to set the repulsion on a portion of the oscillation cycle and adjust the repulsive forces to the minimum that we can register. When scanning in air, achieving this goal is complicated by substantial adhesive forces associated with surface water and/or other adsorbates, which means that the onset of repulsive interaction should be registered against a strong background of attraction. This task can be significantly simplified if the dissipation mode (DM) is used for scanning [5]. In the DM, the probe excitation frequency is maintained equal to its instant resonant frequency (as in FM-AFM), while the signal fed to the *Z*-scanner feedback loop is the oscillation amplitude (as in AM-AFM). The possibilities of using this mode for scanning were discussed in [3–5,35]. A comprehensive theoretical and experimental study of the approach curves was carried out in the framework of the development of the frequency modulation mode [12–14]. When approaching, the probe amplitude in the DM decreases only due to dissipation, changing monotonically (Figure 1a) without any bistabilities typical for conventional AM-AFM. The change in the resonant frequency is non-monotonic, which directly reflects the competing influence of the attractive and repulsive forces for each specific probe and sample. Optimal scanning conditions roughly correspond to the minimum of the frequency-versus-distance curve [5]. By plotting the dependence of the resonant frequency on the amplitude, we can find the value of amplitude *A*_min_ that corresponds to the minimum frequency. The exact value of this amplitude depends on the value of the initial amplitude. With a large initial amplitude *A*_0_, the value of *A*_min_ will be close to *A*_0_, it is difficult to use such an amplitude as a set point, since the slightest instability (decrease) in the level of initial oscillations will lead to disruption of the feedback. For too low *A*_0_, a minimum in the frequency curve is not observed; the frequency decreases monotonically as the probe approaches the sample. The reasons for this are discussed in [5]. The concept of large or small amplitude *A*_0_ is relative, say *A*_0_ = 20 nm may be too large for a sharp tip and too small for a blunt one. The main advantage of DM is the existence of a clear protocol for choosing the optimal scanning parameters for each specific case. It is recommended to start the choice of amplitude *A*_0_ from small values, increasing the amplitude until a minimum in the frequency-versus-distance plot is observed at amplitudes corresponding to 70–90% of the initial amplitude. We then set the amplitude at which the frequency is minimum as the set point *A*_sp_. Further, during the scanning process, we can slightly adjust *A*_sp_, optimizing the image quality. In the DM, along with the surface profile, it is advisable to record the signal of the change in the resonant frequency. It depends on the strength of the tip–sample interaction and often has a better (compared to topography) contrast.

**Figure 4:**
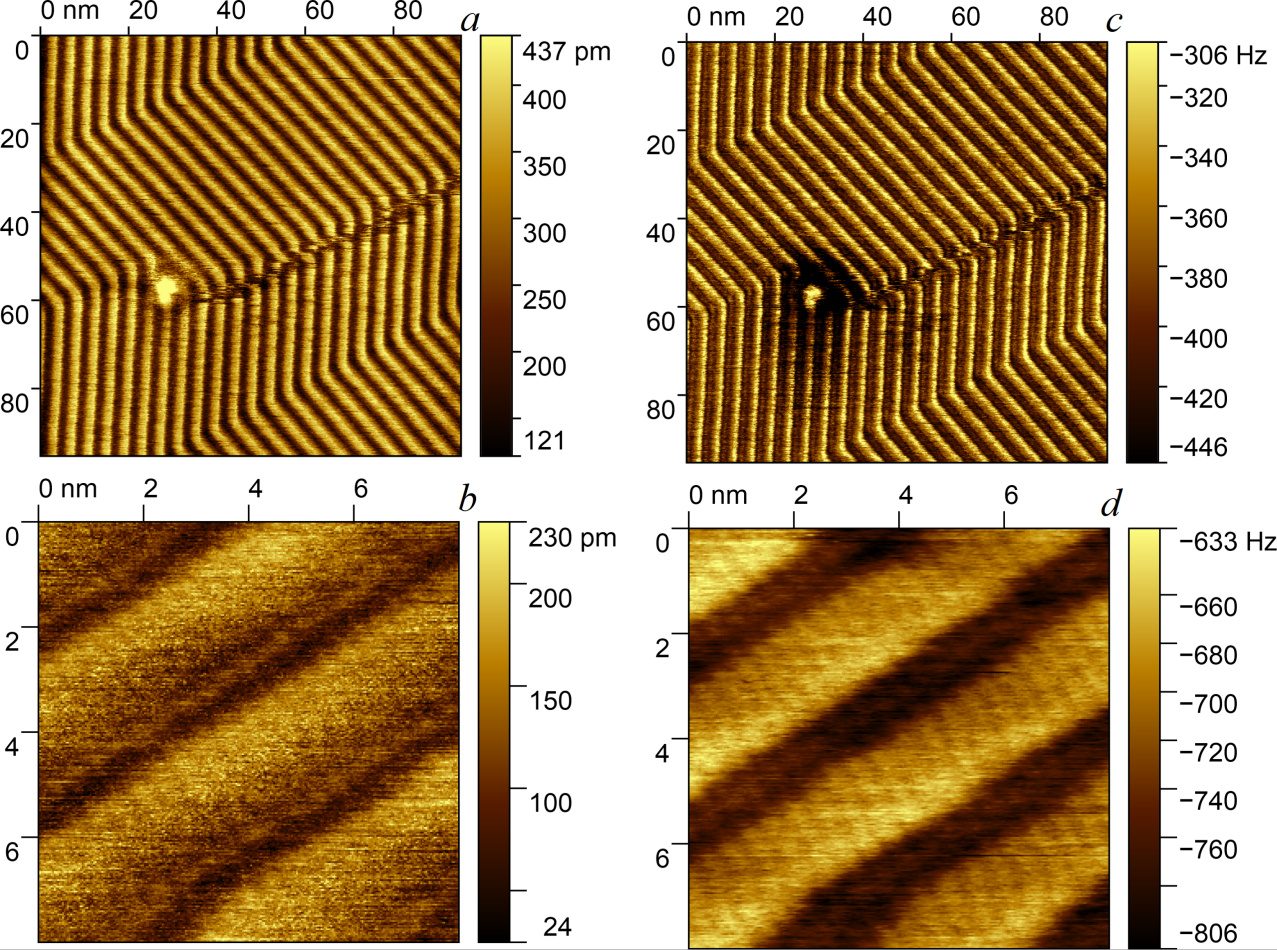
Dissipation mode. Self-organization of palmityl palmitate on graphite. Topography (a, b) and shift of the resonant frequency of the probe (c, d).

Figure 4 shows an example of the use of DM. We investigated the lamellar structure of a self-assembled layer of palmityl palmitate on the surface of highly oriented pyrolytic graphite. The lamellar structure with a period of about 4 nm is clearly visible. It is interesting to note that in this case, when reducing the scanning area, it was possible to see (in the frequency channel) the location of molecular chains with a period of 0.7 nm. Such a high resolution is not typical for scanning under ambient conditions. A standard probe was used, and we were not expecting to have a supersharp tip, though we cannot exclude the possibility that this particular probe happened to be particularly sharp or had a shallow ultrasharp protrusion at the very end. The above procedure for selecting the scanning parameters automatically determined that the probe was very sharp, that is the frequency curve had a minimum at an initial amplitude of several nanometers. Again, this was done before the start of scanning, which significantly reduced the risk of contamination or breaking of the sharp tip as a result of improper selection of the initial amplitude.

It is important that the presence of clear criteria allows one to set the optimal scanning parameters for each specific sample and each probe (the sharpness of which may change during the scanning process). In [36], the lamellar structure was observed even when it was covered with a disordered adsorbate layer. The precise choice of the scanning parameters makes it possible to detect the presence of a lamellar structure with a period of 4–5 nm even with relatively blunt probes, for which a frequency curve minimum appeared at the initial amplitude of the order of 30–40 nm.

Thus, comparing with AM-AFM, we can say that the advantage of DM is a straightforward and accurate procedure for selecting initial scanning parameters, which, in turn, leads to significantly less stringent requirements for the sharpness of the probes used for scanning with high resolution. However, it should be emphasized that these advantages are fully realized on surfaces where, in principle, high lateral resolution is possible. This should be a relatively flat area without protrusions comparable in height to the curvature of the tip. To select a site, it is optimal to use the vertical mode, since it allows one to maintain sharp probes when scanning rough surfaces. In some cases, when it is necessary to detect the presence of a fine structure against the background of larger defects, it is advisable to use a combination of VM and DM. In this case, as in the DM, the self-tuning of the resonance frequency is used. The parameter setting is also similar to that of DM. Height feedback is not used, and scans are performed according to the VM protocol. This combined mode (VDM) can significantly reduce the likelihood of contamination of the probe while maintaining the ability to have high resolution [36]. Nevertheless, it should be noted that it is advisable to use the VDM only in those cases when it is really necessary, that is, when it is necessary to have a lateral resolution of a few nanometers. The vertical mode is more versatile. It is simpler, more reliable, and safer. The VM does not require additional feedback supporting the resonant frequency. In the attractive regime, the weaker interaction of the probe with the sample when using the VM provides better probe safety than the DM. When scanning in liquid, the DM has no advantages. Frequency modulation [35,37–38] or Q-Control [39–40] will be more effective in that case.

## Conclusion

In this paper, we summarized the experience of using the two AFM scanning modes, namely vertical mode and dissipation mode. The measurements were carried out on a SmartSPM atomic force microscope manufactured by AIST-NT (currently produced by HORIBA Scientific) under the control of a modified imaging software that allows for the programming of new procedures and controls. The operation of modern AFMs is based on a digital feedback loop, which provides greater flexibility in the development of new modes. No new electronic elements are required (i.e., oscillators, integrators, or phase-locked loop systems), these tasks are carried out by the software. The introduction of the described techniques into standard AFM control programs is not very difficult, but can significantly simplify operations.

## Supporting Information

File 1 Additional experimental results.
