# Supplementary material for: Two dynamic modes to streamline challenging atomic force microscopy measurements
Source: Beilstein J Nanotechnol. 2021 Nov 15;12:1226–36. doi: 10.3762/bjnano.12.90 (PMC8609242; doi:10.3762/bjnano.12.90)
Supplement: File 1 — Additional experimental results. [file Beilstein_J_Nanotechnol-12-1226-s001.pdf]

BEILSTEIN JOURNAL OF NANOTECHNOLOGY

Supporting Information

for

Two dynamic modes to streamline challenging atomic force microscopy measurements

Alexei G. Temiryazev, Andrey V. Krayev and Marina P. Temiryazeva

Beilstein J. Nanotechnol. **2021,** *12,* 1226–1236. doi:10.3762/bjnano.12.90

Additional experimental results

License and Terms: This is a supporting information file under the terms of the Creative Commons Attribution License (https://creativecommons.org/licenses/by/4.0). Please note that the reuse, redistribution and reproduction in particular requires that the author(s) and source are credited and that individual graphics may be subject to special legal provisions.
The license is subject to the *Beilstein Journal of Nanotechnology* terms and conditions: (https://www.beilstein-journals.org/bjnano/terms)

Examples of using vertical mode to scan tall samples

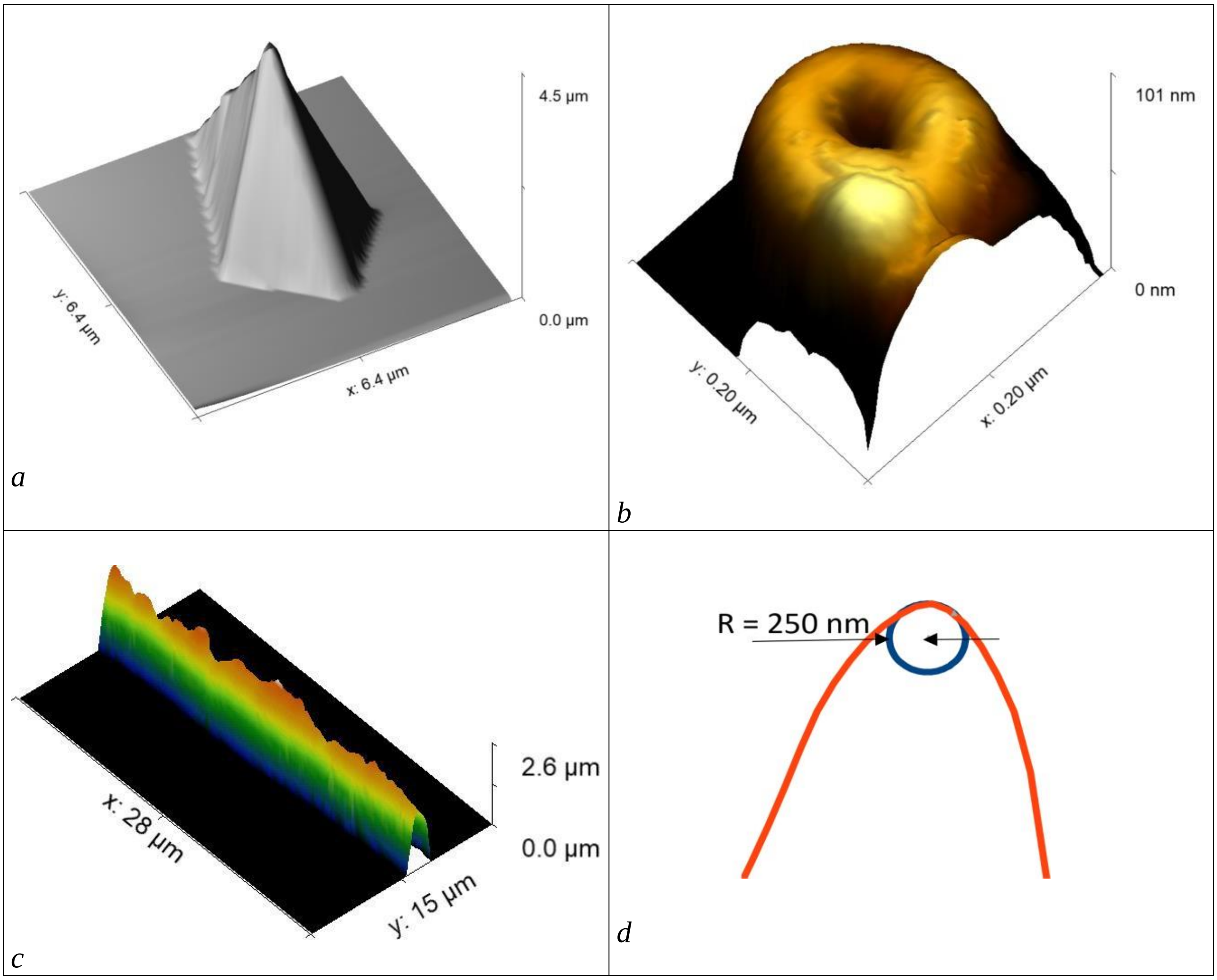

Figure S1: We scanned a micropipette, which is a probe in scanning ion conductance microscopy. Micropipettes are much thinner than the AFM tip cone. A rough scan of a large area gives an image of the AFM probe itself (Figure S1a). To see the end of the micropipette (Figure S1b), we need to scan small area near the highest point.
The VM allows you to scan a razor blade (Figure S1c) and measure its radius of curvature (Figure S1d).

Stability and quality of scanning in vertical mode

Four scans of the rough surface of bismuth telluride were performed at a low free amplitude.

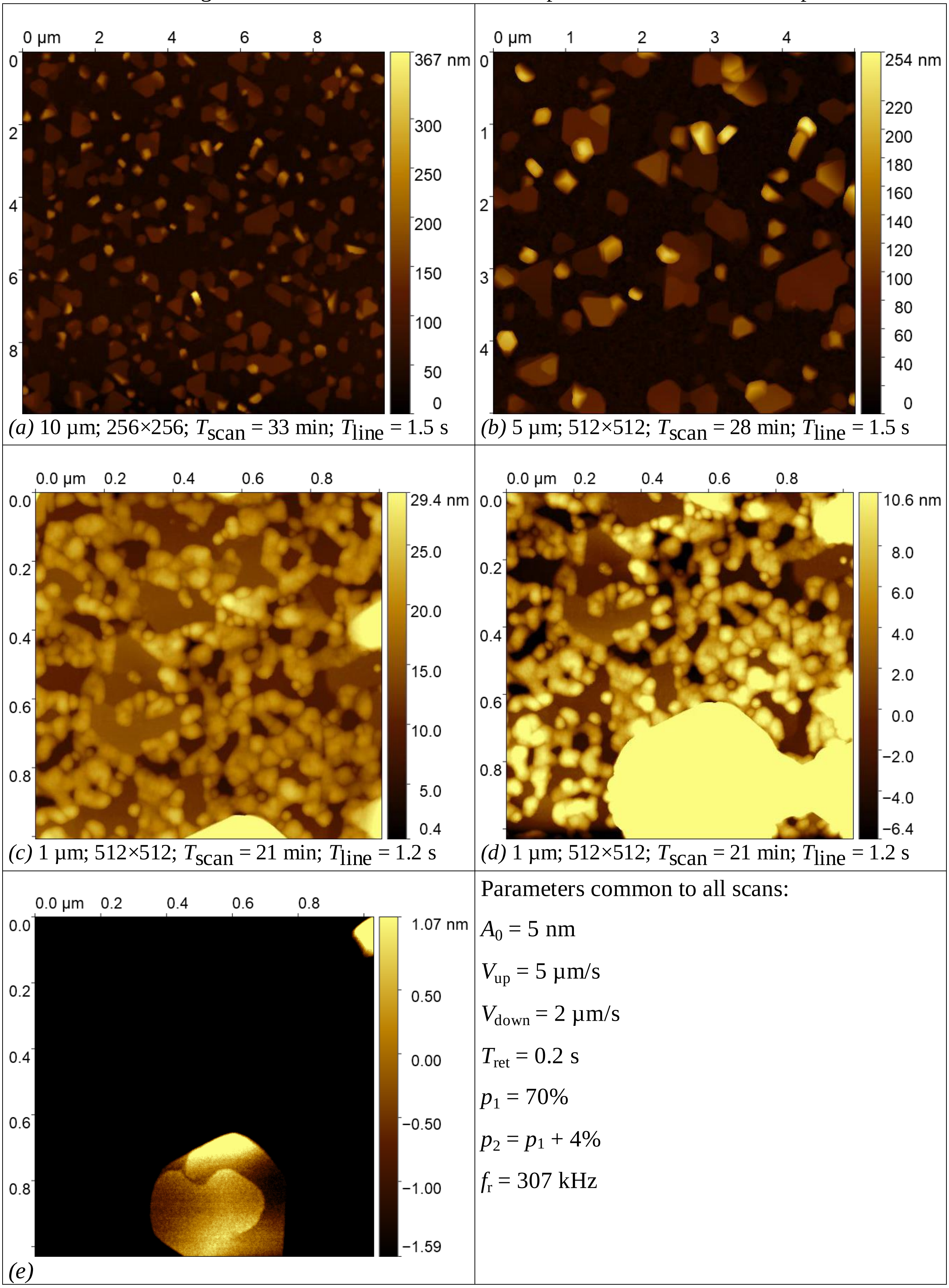

(a) 10 μm; 256×256; T_{scan} = 33 min; T_{line} = 1.5 s

(b) 5 μm; 512×512; T_{scan} = 28 min; T_{line} = 1.5 s

(c) 1 μm; 512×512; T_{scan} = 21 min; T_{line} = 1.2 s

(d) 1 μm; 512×512; T_{scan} = 21 min; T_{line} = 1.2 s

(e)

Parameters common to all scans:

A_0 = 5 nm

V_{up} = 5 μm/s

V_{down} = 2 μm/s

T_{ret} = 0.2 s

p_1 = 70%

$p_2 = p_1$ + 4%

f_r = 307 kHz

Figure S2: (a-d) show 4 consecutive scans; (e) shows the same scan as (d), but the contrast is chosen to show the top of a high crystal. A step with a height of 1 nm is clearly visible.

The scans have no defects on either vertical walls or flat surfaces. No line fitting was applied.

Comparison of vertical mode with standard AM-AFM mode

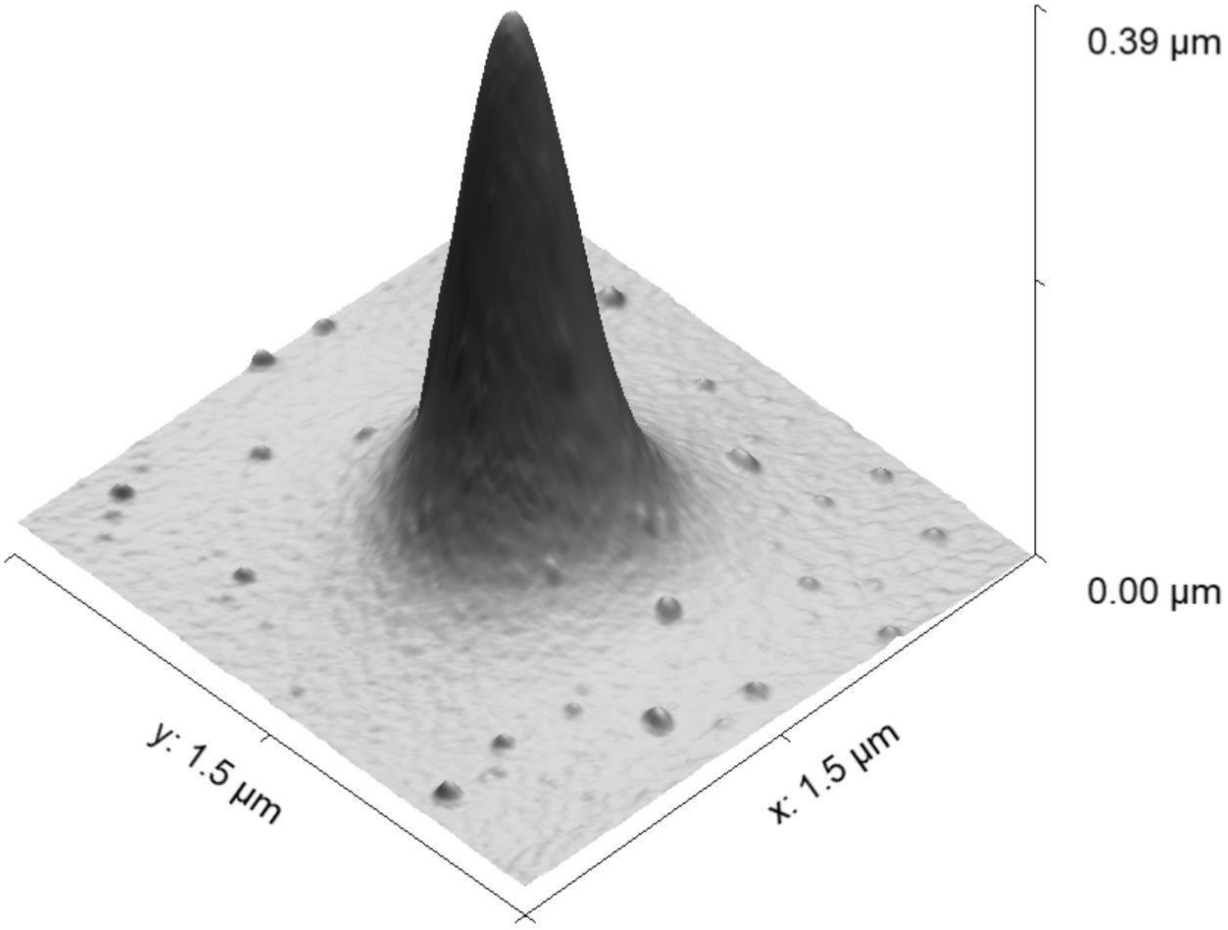

Figure S3: AFM image of the tip on a test structure TGZ 1 (NT-MDT).

We have performed four scans of the same area; 256 by 256 points. They are labeled as A, B, C, D.
A and D have been taken in the *vertical mode* (VM).
B and C – in s*tandard* semi-contact (tapping) mode (SM).
All scans were conducted at free amplitude of 10 nm.
Set point in SM and $p1$ in VM is about 70%, which corresponds to the amplitude of 17 ku (relative units).
For the VM: $p2 = p1 + 4\%$; the return time $T_{ret} = 0.1$ s. $V_{up} = 5$ µm/s; $V_{down} = 2$ µm/s;
A and D differ in T_{line} (time of lateral moving): $T_{line} = 1$ s for A, and $T_{line} = 0.5$ s for D.
B and C differ in scanning rate 0.33 Hz for B (3 s per line) and 1 Hz for C (1 s per line);
We used cantilever Mikromasch NSC15 with nominal force constant 46 N/m and resonance frequency of 328 kHz.

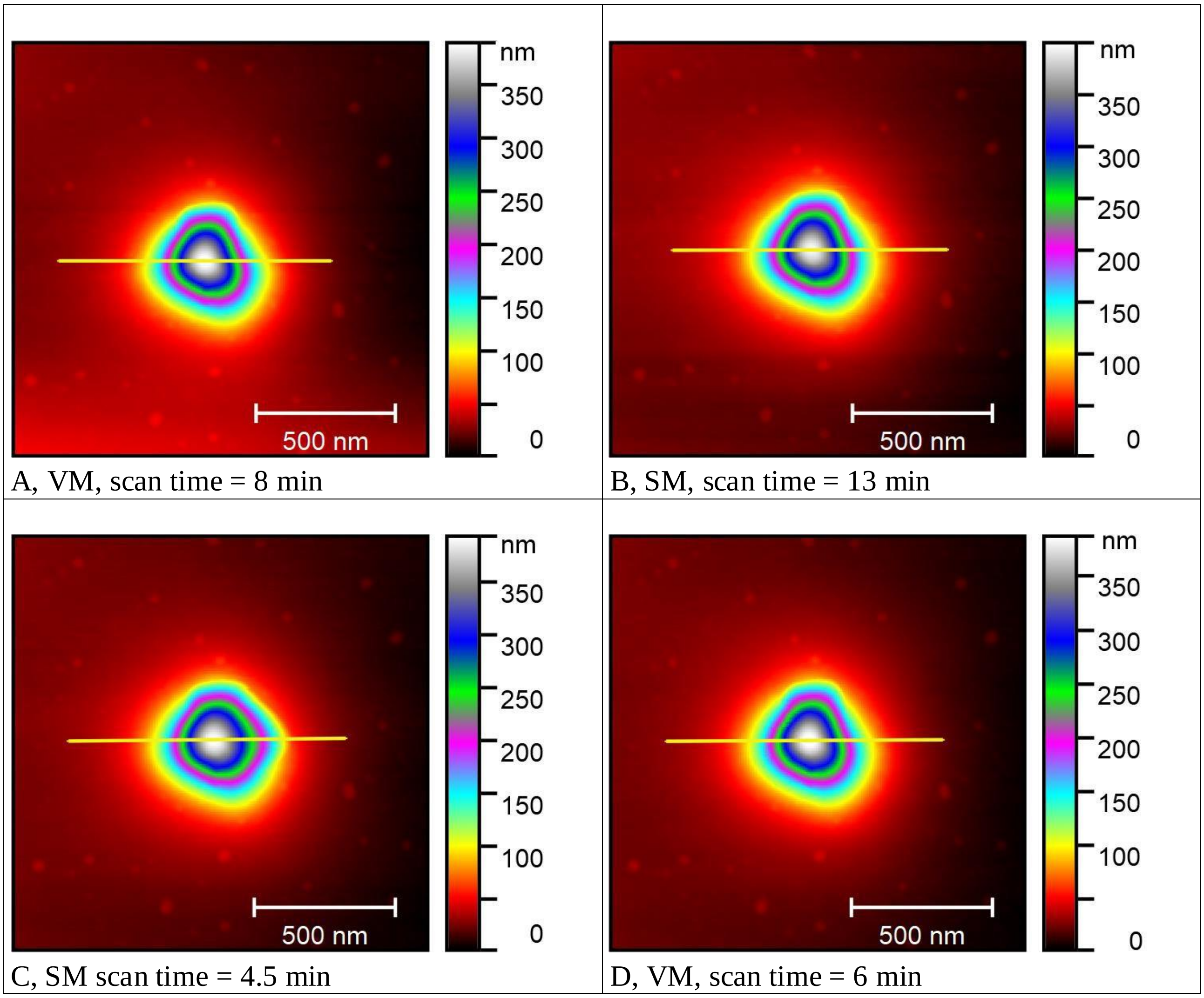

Figure S4: AFM images of the same area in different modes. Scans A, B, D look the same, scan C is slightly different.

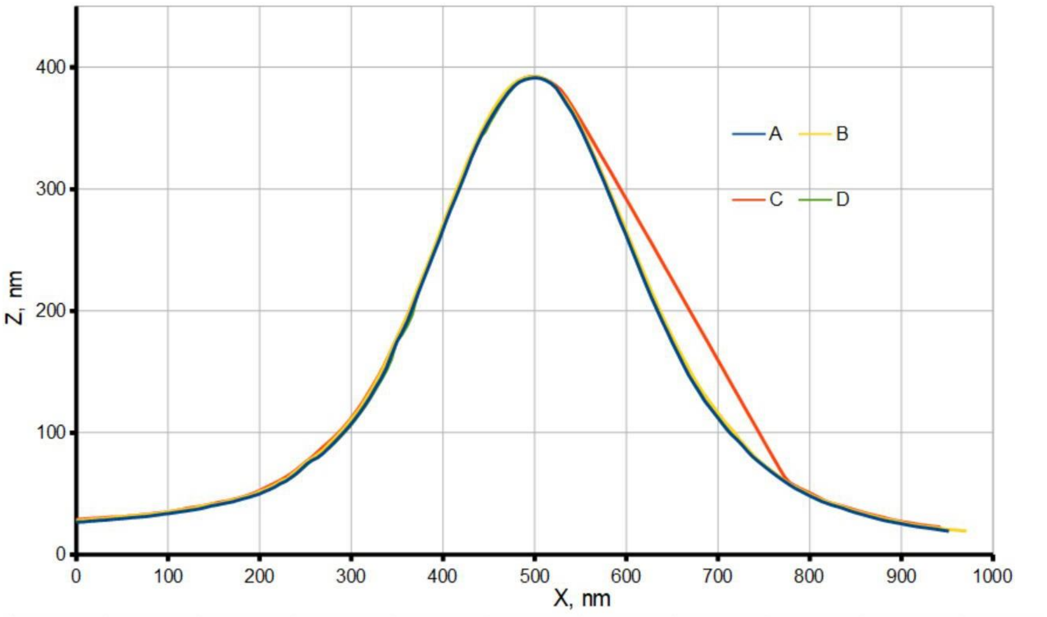

Figure S5: Height profiles along the yellow lines in Figure S4. We can see parachuting in regime C.

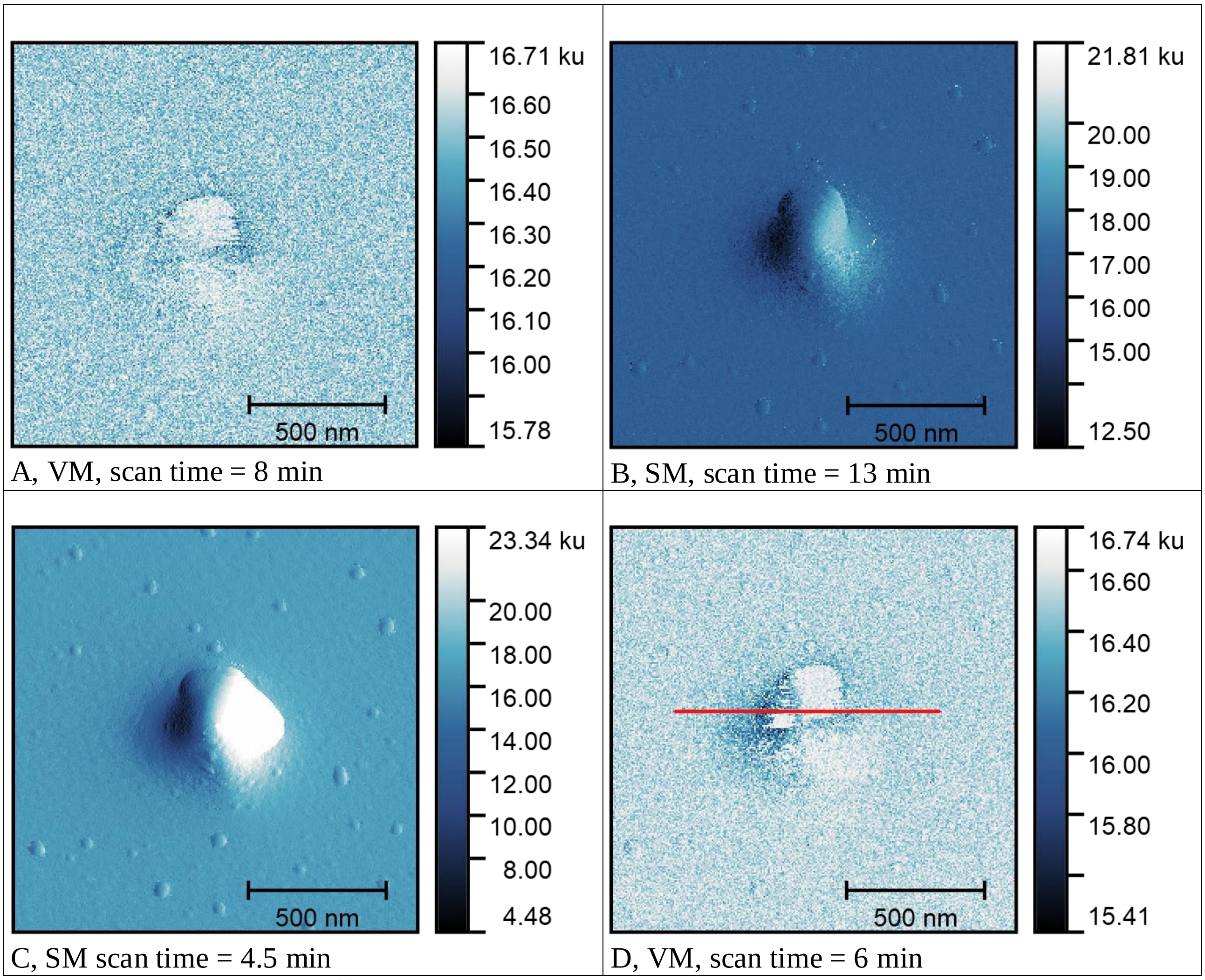

Figure S6: Amplitude in different modes.

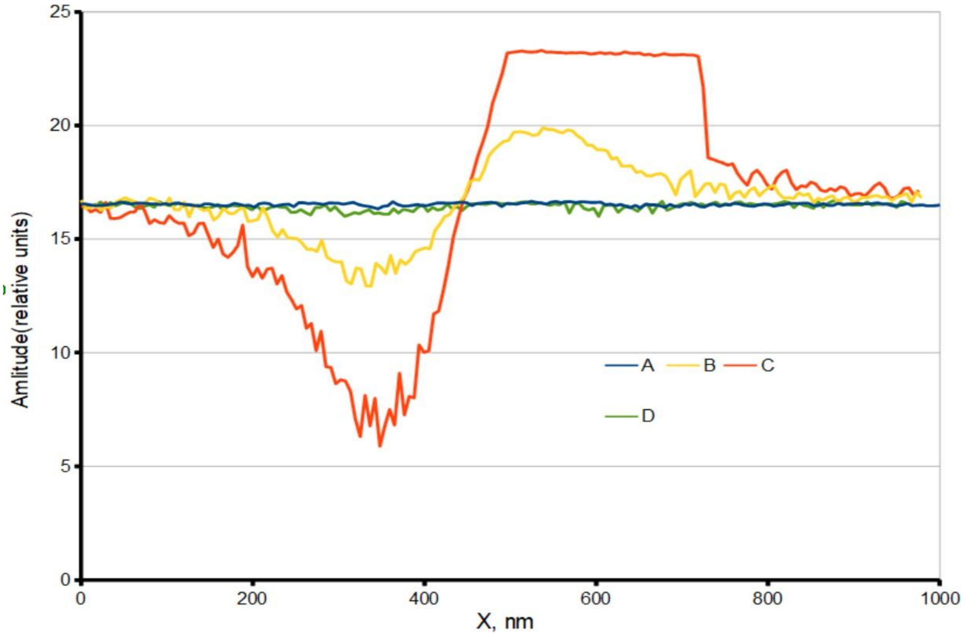

Figure S7: Amplitude variations in different regimes.

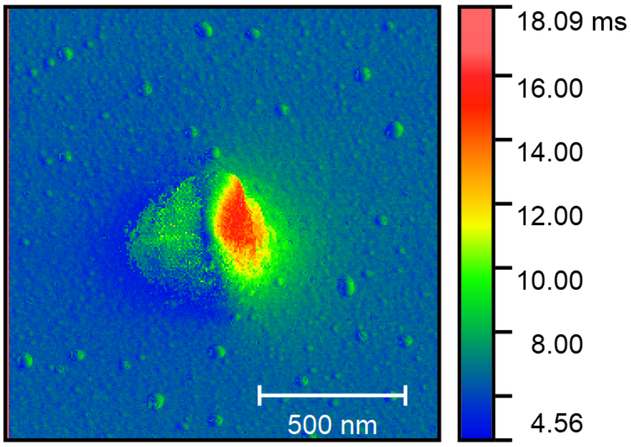

A, VM, scan time = 8 min, T_{line} = 1 s

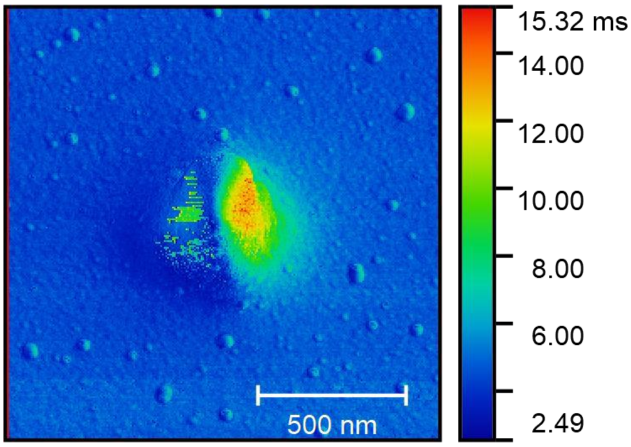

D, VM, scan time = 6 min, T_{line} = 0.5 s

Figure S8: Time to measure at one point for two VM regimes.
At flat areas, the time is about 5.9 ms for A and 4 ms for D. A time for the lateral movement from point to point is 3.5 ms for A, and 1.6 ms for D. Thus, the time for vertical movement in both cases is 2.4 ms.
